# Fluorescent Multiplex Immunohistochemistry Coupled With Other State-Of-The-Art Techniques to Systematically Characterize the Tumor Immune Microenvironment

**DOI:** 10.3389/fmolb.2021.673042

**Published:** 2021-09-21

**Authors:** Anaïs Boisson, Grégory Noël, Manuel Saiselet, Joël Rodrigues-Vitória, Noémie Thomas, Mireille Langouo Fontsa, Doïna Sofronii, Céline Naveaux, Hugues Duvillier, Ligia Craciun, Denis Larsimont, Ahmad Awada, Vincent Detours, Karen Willard-Gallo, Soizic Garaud

**Affiliations:** ^1^ Molecular Immunology Unit, Institut Jules Bordet, Université Libre de Bruxelles, Brussels, Belgium; ^2^ IRIBHM, Université Libre de Bruxelles, Brussels, Belgium; ^3^ Flow Cytometry Facility, Institut Jules Bordet, Université Libre de Bruxelles, Brussels, Belgium; ^4^ Department of Pathology, Institut Jules Bordet, Université Libre de Bruxelles, Brussels, Belgium; ^5^ Oncology Medicine Department, Institut Jules Bordet, Université Libre de Bruxelles, Brussels, Belgium

**Keywords:** tumor immune microenvironment, breast cancer, tumor-infiltrating lymphocytes, tertiary lymphoid structure, fluorescent multiplex immunohistochemistry

## Abstract

Our expanding knowledge of the interactions between tumor cells and their microenvironment has helped to revolutionize cancer treatments, including the more recent development of immunotherapies. Immune cells are an important component of the tumor microenvironment that influence progression and treatment responses, particularly to the new immunotherapies. Technological advances that help to decipher the complexity and diversity of the tumor immune microenvironment (TIME) are increasingly used in translational research and biomarker studies. Current techniques that facilitate TIME evaluation include flow cytometry, multiplex bead-based immunoassays, chromogenic immunohistochemistry (IHC), fluorescent multiplex IHC, immunofluorescence, and spatial transcriptomics. This article offers an overview of our representative data, discusses the application of each approach to studies of the TIME, including their advantages and challenges, and reviews the potential clinical applications. Flow cytometry and chromogenic and fluorescent multiplex IHC were used to immune profile a HER2+ breast cancer, illustrating some points. Spatial transcriptomic analysis of a luminal B breast tumor demonstrated that important additional insight can be gained from this new technique. Finally, the development of a multiplex panel to identify proliferating B cells, Tfh, and Tfr cells on the same tissue section demonstrates their co-localization in tertiary lymphoid structures.

## Introduction

The tumor immune microenvironment (TIME) plays a critical role in cancer development, progression, and treatment responses. It is defined by the immune cells, antigens, and soluble factors (including cytokines, chemokines, and immunoglobulins) that surround and influence tumor cells. The molecular and cellular composition of the TIME influences disease outcome via the balance between pro- and anti-tumor innate and adaptive immune responses. Human tumor-infiltrating lymphocytes (TILs) such as CD8^+^ cytotoxic T cells, conventional CD4^+^ T cells, T follicular helper cells (T_FH_) (van der Leun et al., 2020), B cells (Wouters and Nelson, 2018), and natural killer cells (Stabile et al., 2017) are generally associated with favorable (anti-tumor) immune responses, together with γδ T cells (Lo Presti et al., 2020) and eosinophils (Grisaru-Tal et al., 2020). Many studies also show that tumor-associated macrophages and neutrophils, myeloid-derived suppressor cells, and regulatory T (Treg) cells are key drivers of cancer progression via their ability to promote tumor cell functions such as proliferation, aggressiveness, and dissemination in parallel with suppression of T cell-mediated anti-tumor immunity (Lecot et al., 2019; Ohue and Nishikawa, 2019; Davidov et al., 2020). A caveat is that immune cells are functionally heterogeneous and plastic with most capable of divergent behavior based on their activation status and the surrounding microenvironment.

Beyond the TIME composition, studying the location and spatial distribution of immune cells can provide a framework for understanding tumor biology and identifying potential predictive biomarkers. Spatial characteristics of tumors can be initially stratified based on tissue architecture such as intratumoral, peritumoral (or stromal) areas, and the invasive margin. In human breast cancer (BC), both intratumoral and stromal TIL have been consistently and significantly associated with overall survival (OS) in the HER2^+^ and triple-negative subgroups (Dieci et al., 2015; Hendry et al., 2017). Recent studies of TIL subsets in BC revealed stromal CD3^+^ T cells and FOXP3^+^ Treg were associated with disease-free survival (DFS) but not their intratumoral counterpart while both intratumoral and stromal CD8^+^ cytotoxic T cells predict longer DFS (Koletsa et al., 2020). Recent studies of tumor and immune spatial distribution at the single-cell level demonstrated a significant correlation with disease outcomes. For example, T cells and proliferating tumor cells were found in close proximity in immunoedited colorectal cancer metastases whereas short distances were seen between T cells and PD-L1^+^ cells in non-immunoedited metastases (Angelova et al., 2018). Analysis of matched primary and recurrent head and neck squamous cell carcinoma detected CD8^+^ T cell exclusion from tumor nests and close proximity between Treg or myeloid cells with tumor cells at relapse (Banik et al., 2020). TIL in the invasive margin or stroma can form tertiary lymphoid structure (TLS), which are similar to secondary lymphoid organs with a T cell zone adjacent to a B cell follicle that contains germinal center B cells, T_FH_ cells (Garaud et al., 2019), and mature dendritic cells (Dieu-Nosjean et al., 2008). A TLS presence is associated with favorable clinical outcomes (Dieu-Nosjean et al., 2008; Silina et al., 2018) and responses to immune checkpoint blockade (Cabrita et al., 2020; Helmink et al., 2020; Petitprez et al., 2020). The TLS maturation stage also harbors important prognostic information on the risk of disease recurrence (Posch et al., 2018; Silina et al., 2018). This means that deeper compositional and spatial analysis of immune cells infiltrating the tumor is needed to achieve a better understanding of effective anti-tumor immunity and discover new potential biomarkers.

Recent technological advances for phenotypic and transcriptional analysis of individual cells in the context of their spatial distribution are new, powerful tools for studying the TIME and identifying potential biomarkers. Fluorescent multiplex immunohistochemistry (mIHC) can simultaneously evaluate multiple biological markers on a single formalin-fixed, paraffin-embedded (FFPE) section. The objective of this review is to comparatively evaluate mIHC relative to more established TIME analytical techniques. We will consider their relative strengths and limitations as well as use our laboratory’s studies of the BC immune microenvironment (as an example for other solid tumors) to showcase how mIHC can help to generate a more complete picture.

### Tumor Immune Microenvironment Evaluation Using Fresh Specimens

Archival FFPE blocks are the most readily available source of tissue samples for translational research but fresh and frozen samples, including biopsies and surgical tissue specimens, are increasingly being collected for tumor microenvironment analysis. Our laboratory developed a methodology for the rapid isolation of intact lymphoid cells from normal and abnormal tissues in an effort to evaluate them proximate to their native state (Garaud et al., 2014). Briefly, the tissue is mechanically dissociated without enzymatic digestion to prepare single-cell suspensions. Lymphoid cells can be easily used for cell sorting, isolation, cryopreservation, and/or phenotypic analysis. Additionally, because this is an enzyme-free method, the primary tissue supernatant from the homogenates can be used to characterize and compare cytokines, chemokines, immunoglobulins, and antigens present in normal and malignant tissues (Garaud et al., 2018; Garaud et al., 2019).

#### Flow Cytometry

**FIGURE 1:**
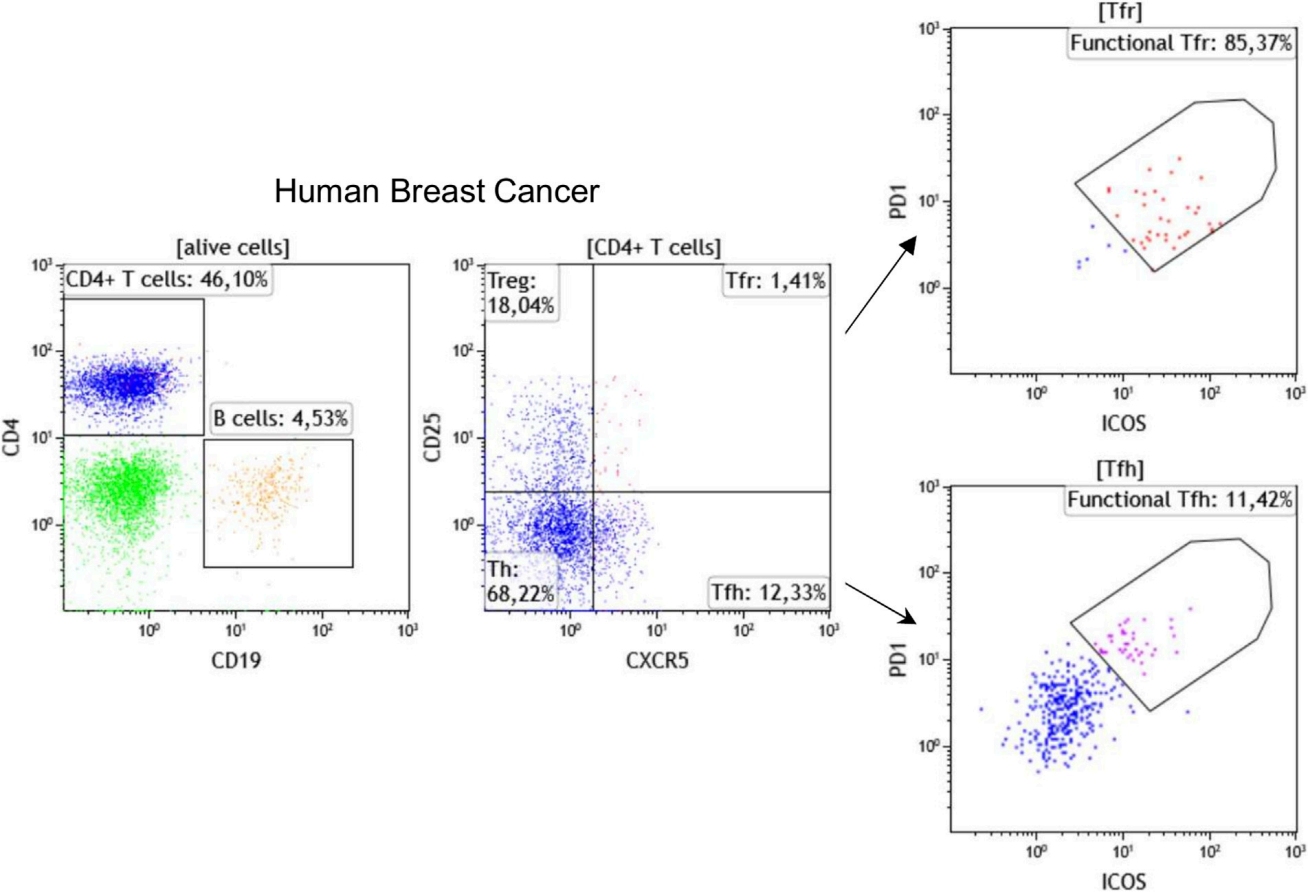
Immunophenotyping of T_FH_ and T_FR_ cells by flow cytometry using fresh breast cancer tissues. Representative dot plots show the percentage of CD19^+^ B cells, CD4^+^ T cells, CD25^−^CXCR5^−^CD4^+^ helper T cells (Th), CD25^+^CXCR5^−^CD4^+^ regulatory T (Treg), CD25^+^CXCR5^+^CD4^+^ T_FR_, and CD25^−^CXCR5^+^CD4^+^ T_FH_ cells. The functionality of T_FH_ and T_FR_ cells has been demonstrated to be linked with PD-1 and ICOS expression levels.

Flow cytometry is a broadly applied, reliable technique for quantitative and qualitative multi-parametric analysis of single cells in solution. Traditional flow cytometers can detect up to 20 parameters (size, granularity, and 18 fluorescent detectors); however, advances in fluorochromes and instrumentation now make it possible to perform experiments with 30 + parameters. In oncoimmunology, flow cytometry has been used for years to routinely classify hematological malignancies via the analysis of immune subpopulations using lineage markers, including T cell markers (CD3, CD4, CD8), B cell markers (CD19, CD20), monocyte markers (CD14, CD11b), and NK cell markers (CD56, CD161) in parallel. Flow cytometry using these and other markers including those related to immune cell differentiation, maturation, activation, functionality, and antigen specificity are now also used to characterize the TIME. Our laboratory has established >30 panels, each with up to 10 fluorescent markers, specifically designed for flow cytometric analysis of immune subpopulations in blood and tissues from cancer patients. In addition, we use flow cytometry to identify the most reliable markers to be tested for mIHC. A recent example is our addition of CXCR5, the CXCL13 receptor, which is an important TLS chemokine, to classical lineage markers for the characterization of TLS-associated lymphoid cells (Noël G., in press). Figure 1 shows a representative strategy for flow cytometric immunophenotyping of CD3^+^CD4^+^CXCR5^+^CD25^−^ T_FH_ and CD3^+^CD4^+^CXCR5^+^CD25^+^ follicular regulatory T (T_FR_) TIL in fresh BC tissue. Furthermore, the active state of these specialized CD4^+^ T cell subpopulations can be achieved using PD-1 and ICOS expression levels to identify functional PD-1^hi^ICOS^int^ T_FH_ and functional PD-1^int^ICOS^hi^ T_FR_ TIL (Shi et al., 2018; Xing et al., 2020; Noël G., in press). These flow cytometry data will be used to build our chromogenic and fluorescent mIHC panels.

#### Multiplexed Bead-Based Immunoassays

Immune cells and their soluble mediators, including cytokines, chemokines, and immunoglobulins, are key players in human tumor progression and can be numerically and functionally altered both in the periphery and in the tumor microenvironment. Immunoassays are exemplified by the widely used ELISA, one of the most commonly used techniques for detecting soluble mediators. The development of multiplexed bead-based immunoassays has led to their emergence as a new standard for detecting and quantifying a broad variety of immune mediators using small amounts of blood or bodily fluid samples. This approach is based on fluorescent microspheres (beads) in a sandwich immunoassay, which can simultaneously detection up to 500 markers (depending on system design) using a dual-laser analytical flow cytometer. Soluble mediators in the TIME can be effectively analyzed using multiplex bead-based immunoassays to examine primary tumor tissue supernatants from the homogenates (Garaud et al., 2014; Garaud et al., 2018; Garaud et al., 2019; Ray et al., 2020; Autenshlyus et al., 2021).

#### Challenges

The greatest challenge in examining TIL in fresh tumor samples is access to sufficient quantities of tissue for flow cytometric analysis. Additionally, these analyses can yield inconsistencies depending upon how the tissues are handled during the pre-analytical phase, which can be affected by a variety of parameters including the length of time from biopsy/surgery to sample preparation, ischemia, temperature, and storage. Preparation of fresh tissues can also be limited by the availability of specialized equipment, including a vertical laminar flow hood, a tissue dissociator, and/or a flow cytometer. Thus, at the present time, this approach remains a research tool that needs to evolve further before it can be considered for routine clinical practice, as currently done for hematological malignancies.

Although multiplexed flow cytometry can facilitate detailed characterization of TIME complexity, this approach does not provide information on spatial relationships. This can be achieved by using complementary approaches in parallel such as conventional or mIHC to examine TIL organization and distribution within the TIME.

### Tumor Immune Microenvironment Evaluation Using Formalin-Fixed, Paraffin-Embedded Tissue Specimens

FFPE tissue blocks are widely prepared in the routine pathology lab for chromogenic IHC (cIHC) staining as a part of diagnostic testing. Pathology departments archive vast numbers of FFPE blocks, but currently comparatively few frozen tissues, making the former a readily available resource for studying biomarkers.

#### Chromogenic Immunohistochemistry

**FIGURE 2:**
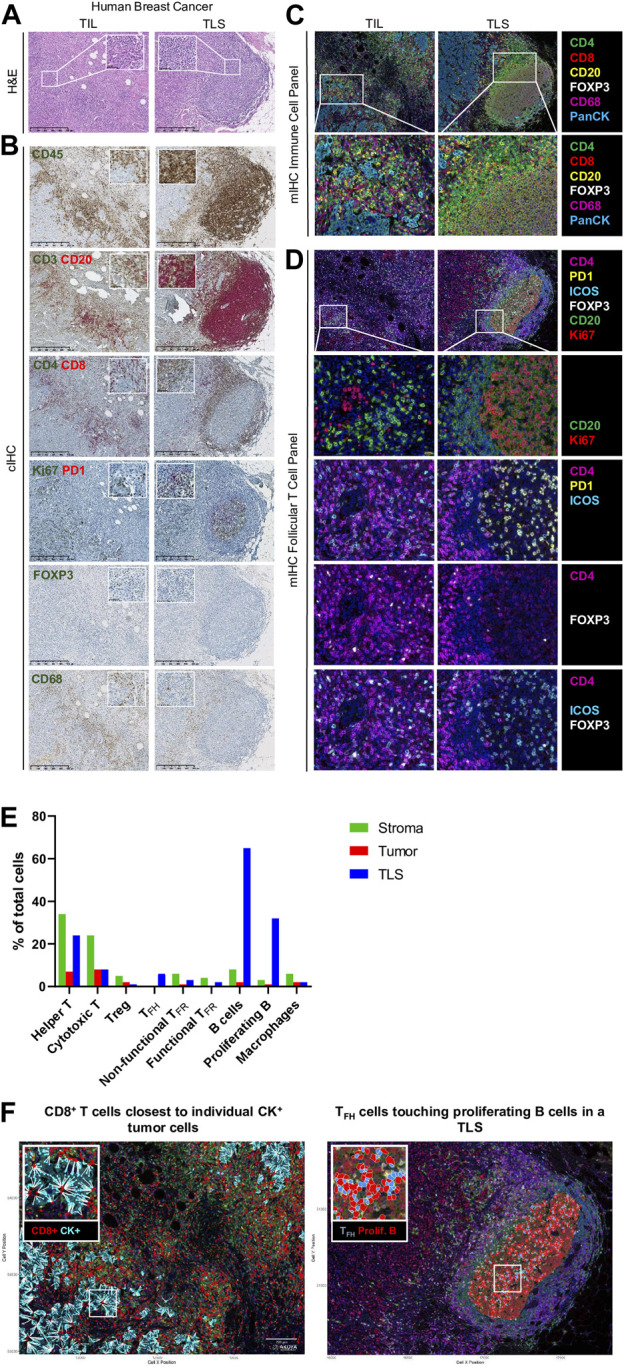
Immune profiling of breast cancer. **(A–D)** Representative images of tumor-infiltrating lymphocyte (TIL) and tertiary lymphoid structure (TLS) in FFPE breast cancer sections detected by **(A)** hematoxylin and eosin staining (H&E), **(B)** chromogenic IHC (cIHC), and **(C, D)** multiplex IHC (mIHC). **(E)** Quantification of immune cells in stromal, tumoral, and TLS areas using InForm and PhenoptrReports. **(F)** Spatial distribution of immune cells including CD8^+^ T cells closest to individual CK^+^ tumor cells and T_FH_ cells touching proliferating B cells in a TLS. H&E and cIHC slides were scanned at ×40 magnification and the images are displayed at ×5 and ×40 magnification. mIHC slides were scanned at ×20 magnification.

Assessment of the immune infiltrate in diverse solid tumor types on hematoxylin and eosin- (H&E-) stained tissue sections is widely used for diagnosis and to provide prognostic and predictive information. TIL evaluation for early BC was the first to be recommended for routine characterization and reporting at the St. Gallen Consensus Conference 2019 (Balic et al., 2019). This analysis is based on a standardized method established by the International Immuno-Oncology Biomarkers Working Group (Hendry et al., 2017). While H&E staining is suitably reproducible and accurate for global TIL scoring (Figure 2A), our previous data revealed its lack of accuracy and reproducibility for TLS assessment (Buisseret et al., 2017a). Additionally, H&E-stained tissues do not provide any information on immune subsets in TIME.

CIHC is a relatively easy, inexpensive, and established technique based on antibody-mediated target antigen recognition that is detected using enzymes, such as horseradish peroxidase (HRP) or alkaline phosphatase (AP) to catalyze a color-producing reaction. Most frequently, detection is done using the 3, 3′-diaminobenzidine (DAB) chromogen, which precipitates in brown. Single detection methods are most commonly used to identify a particular biomarker of interest; however, the availability of new chromogens has led to the development of dual, triplex, and multiplex staining when the target antigens are not on the same cells or subcellular localization (i.e., cell membrane and nucleus). Studies of the BC TIME in our lab were facilitated through the development of numerous single and dual cIHC panels (Gu-Trantien et al., 2013; Buisseret et al., 2017b; Solinas et al., 2017). Dual cIHC is based on two consecutive stains of a single tissue section using DAB followed by AP Red detection on the BenchMark XT autostainer (Ventana Medical Systems) (Buisseret et al., 2017b). An example of a BC immune infiltrate, previously analyzed as fresh tissue by flow cytometry and stained by various single and dual cIHC markers, is shown in Figure 2B. These images reveal the ready global detection using dual IHC for the majority of TIL with CD3 and CD20 or CD4^+^ and CD8^+^ T cell distribution as well as the location of CD20^+^ B cells and CD68^+^ macrophages and the organization of TIL in TLS. The advantages of using various cIHC panels include the preservation of tissue antigenicity, the automated process, and the ready visualization of DAB and Red precipitates using a brightfield microscope. The main limitations of this approach are the limited number of targets evaluated and the depletion of multiple tissue sections for single or dual marker analysis. More recently, multiplexed methodologies have gained popularity because they identify many biomarkers on the same tissue section simultaneously. These approaches include sequential immunoperoxidase labeling and erasing (SIMPLE) using alcohol-soluble peroxidase substrate 3-amino-9-ethylcarbazole combined with an antibody-antigen dissociation (Glass et al., 2009) and multiplexed consecutive IHC-staining on a single slide (MICSSS) that employs iterative cycles of tagging, image scanning, and destaining of the chromogenic substrate on a tissue section (Remark et al., 2016), which can visualize up to five or ten markers, respectively.

#### Immunofluorescence

Immunofluorescent (IF) techniques rely on antibodies tagged with a fluorescent dye to label antigens via their recognition and binding to specific epitopes. Direct and indirect IF are routinely employed, with direct detection done via a fluorophore-primary antibody conjugate and indirect detection requiring first recognition by the primary antibody followed by a fluorophore-conjugated secondary antibody directed to it. The advantage of direct IF is the rapidity of a single step that permits simultaneous staining with numerous antibodies from the same species. Indirect IF on the other hand has the advantage of higher sensitivity via the signal amplification generated by using secondary antibodies but is limited by the necessity to use antibodies from different species. Direct and indirect IF can be combined to amplify the signal for weaker targets and stain multiple primary antibodies from the same species concurrently.

#### Fluorescent Multiplex Immunohistochemistry

Fluorescent mIHC has developed into a feasible approach as a result of technological advances and cIHC/IF limitations. The simultaneous detection of multiple markers on a single section provides a comprehensive view of tissue composition, cellular functionality, subpopulation densities, and cell-cell interactions, to name a few, and is helping to drive mIHC development. Among different mIHC approaches, the Perkin Elmer/Akoya Biosciences Phenoptics™ system is currently capable of detecting up to eight biomarkers plus DAPI (nuclear cell counterstain). This system is based on sequential staining using tyramide signal amplification (TSA) to increase the signal 10-times more than conventional IHC (Faget and Hnasko, 2015). Further, the fluorescent deposit is covalently bound to tyrosine residues on or immediately surrounding the target epitope via activation of the tyramide by the HRP conjugated secondary antibody. This covalent bond enables both primary and secondary antibodies to be stripped from the tissue section via successive rounds of heat treatment (microwave, water bath, steamer, HIER platform, etc.), which has the added benefit of limiting antibody cross-reactivity and non-specific staining. The advantages of fluorescent mIHC include detection of low abundant proteins and using antibodies from the same species.

Our lab currently uses the Vectra® Polaris™ Automated Quantitative Pathology Imaging System for acquisition, which allows the visualization, analysis, quantification, and phenotyping of immune and other cells *in situ* via the integrated inForm and phenoptr/phenoptrReports tissue analysis software packages (Akoya Biosciences®). Multispectral acquisition can also be performed using a Zeiss LSM confocal microscope equipped with a PMT spectral 34-channel QUASAR (Carl Zeiss). The advantages of Akoya’s platform include the unmixing of overlapping fluorophore emission spectra when using the spectral library containing each fluorophore employed, subtraction of tissue auto-fluorescence, and a fully integrated workflow. Furthermore, phenoptrReports provides the quality of the unmixing spectral library, signal strength, and crosstalk, which enables researchers to more readily optimize their multiplexed staining assays for best-in-class quantitative analysis. Additional stand-alone image analysis software packages are also available that can analyze multispectral images, including the HALO® Image Analysis Platform (Indica Labs), Visiopharm’s AI-powered Phenotyping module (VISIOPHARM®), QuPath (Bankhead et al., 2017), and ImageJ. We developed our own mIHC panels to better characterize the BC TIME by testing various commercially available antibodies for optimal labeling of immune cell subpopulations. The first panel is used to locate the major T cell subpopulations, B cells, and macrophages (Figure 2C). Consecutive FFPE BC tissue sections from tumors previously analyzed using flow cytometry (fresh tissue) and cIHC (FFPE) are stained manually. The multispectral images for major immune subpopulations show massive stromal infiltration by CD4^+^ and CD8^+^ TIL together with CD20^+^ TIL-B and CD68^+^ macrophages in association with a TLS (Figures 2C,E). Another panel was designed to detect CD4^+^ follicular helper subpopulations in TLS, which includes functional T_FH_ TIL (PD-1^hi^ICOS^+^, non-functional T_FH_ TIL are PD-1^lo/int^ICOS^lo^), functional T_FR_ TIL (ICOS^+^FOXP3^+^), and non-functional T_FR_ TIL (ICOS^−^FOXP3^+^) together with Ki67^+^CD20^+^ proliferating TIL-B (Figures 2D,E) (Noël G., in press). Proliferating B cells and T_FH_ were not observed in TIL outside of a TLS. The spatial distribution of these immune cells was analyzed using phenoptrReports to identify CD8^+^ TIL nearest to CK^+^ tumor cells and T_FH_ TIL touching proliferating B cells in the TLS (Figure 2F). All of the mIHC data we generated were consistent and complementary with our FACS and cIHC data for the same tumor in terms of immune cell detection, activation status, and localization within the BC TIME.

#### Challenges

While tissue imaging is widely used to investigate immune cell phenotypes and their spatial relationships, its principal limitations are the restricted number of targets analyzed on a single slide and the dynamic range of marker intensity. For the latter, protein must be expressed above a minimal threshold and then scoring is based on the presence or absence of markers or a semi-quantitative H-score. TSA-based reagents for mIHC are more advantageous in this regard because they amplify the signal intensity and covalently bind the fluorophore to the target; however, there is still a risk of interference. TSA interference can derive from overactive tyramide deposits leading to a reduction or inhibition of antigen recognition via steric hindrance (umbrella effect) and/or tyrosine depletion, particularly when two or more markers are at the same cellular site [26]. Spectral bleeding, an artifact where the signal from one channel interferes with the channel being imaged, leads to false-positive staining and occurs between spectrally proximate fluorophores if the signal intensity is not well balanced [26]. In addition, the acquisition and characterization of mIHC images require both a multispectral imaging system and image analysis software. Research efforts to overcome these limitations include using DNA-barcoded antibodies such as the InSituPlex® Technology (ULTIVUE), the CODEX® system (AKOYA Biosciences®), and Digital Spatial Profiling (DSP) technologies (NanoString®). Tissue management, fixation procedures, storage conditions, and sectioning can also affect staining. Multiplex panel development thus requires optimization and validation (detailed below) to produce reproducible, reliable, and high-quality stained tissues.

Beyond technical limitations, image acquisition and analysis need to be standardized to reduce the likelihood of misinterpretation. First, whole-slide analysis, excluding necrotic areas, normal tissues, or vessels, should be favored whenever possible. The region of interest should be confirmed by a trained pathologist and fully cover individual fields for analysis. For larger tissues, the size of data tables can be reduced by randomly placing individual fields on a grid covering 50% or 25% of the region of interest using the phenochart viewer. Second, image analysis using mIHC provides information about the spatial organization including proximity between different types of cells; however, these analyses can be affected by the density of cells. Recent studies of the immune microenvironment during metastatic progression revealed shorter distances between T cells and proliferating tumor cells in immunoedited metastasis compared to unedited metastasis (Angelova et al., 2018). In addition, the tumor compartment should be taken into account in analysis such as the invasive margin or the center of the tumor as immune infiltration varies between these areas. To overcome this limitation, proximity analysis should be performed in tissues/areas with similar cell densities.

## Development of a Multiplexed Panel for T Follicular Helper Cells

Our active development of mIHC panels for our studies has highlighted the essential factors one needs to consider when optimizing and validating a panel that includes three markers expressed at the membrane on the same cellular subpopulation. As an example, we describe our panel for characterizing follicular cells in TLS, which includes proliferating B cells, T_FH_, and T_FR_ cells stained manually with antibodies to CD4, PD-1, ICOS, FOXP3, CD20, Ki67, and DAPI on FFPE sections. Multispectral images were acquired on a Vectra® Polaris™, analyzed with InForm software, and the quality report and marker quantification were generated with the phenoptrReports package.

### Monoplex Assay Development

The first step in the development of an mIHC panel is to define the proper staining parameters for a single antibody and Opal pair using monoplex slides. Library slide development and primary antibody optimizations for antigen retrieval, titration, epitope sensitivity, and antibody stripping efficiency will be not addressed here as they are detailed in Akoya Biosciences® development guide and common for mIHC panel development. Human tonsil and BC FFPE tissue sections were used in the monoplex assays while multiplex assays were performed only on BC tissue sections.

#### Pairing Opal Fluorophores With Primary Antibodies

**FIGURE 3:**
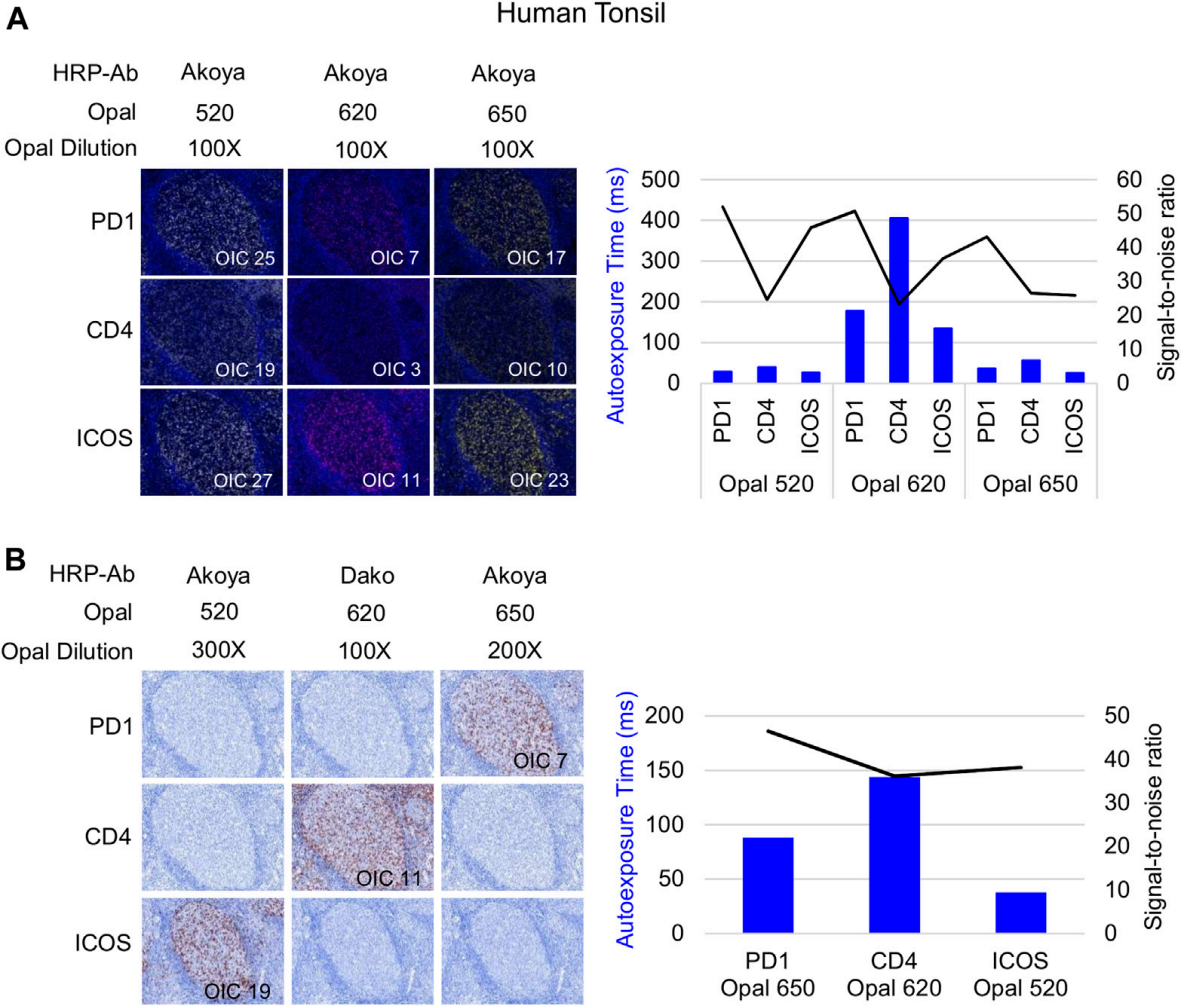
Antibody-Opal pairing and signal balance of three markers co-localized on the cell surface. **(A)** Composite images of consecutive monoplex FFPE tonsil sections stained with PD-1, CD4, or ICOS with Opal 520, 620, or 650. The graph shows the autoexposure times (blue bars) and the signal-to-noise ratio (black curve) for each pairing. The signal-to-noise ratio was calculated via dividing the Opal intensity count (OIC) by the Opal background obtained in InForm. **(B)** Simulated DAB IHC images of optimized monoplex staining in **(A)**. The graphic shows the autoexposure times (blue bars) and the signal-to-noise ratio (black curve) of optimized monoplex staining determined by InForm. The slides were scanned at ×20 magnification with the Vectra Polaris and the composite images were analyzed with InForm software (v.2.4.8) and PhenoptrReports (Kent S Johnson (2020). phenoptr: inForm Helper Functions. R package v.0.2.6. https://akoyabio.github.io/phenoptr/).

Following Akoya’s recommendation, the pairing of an Opal fluorophore with an individual marker necessitates accounting for the Opal’s brightness on the Vectra Polaris scanner (Table 1). Low marker expression should be assigned a brighter fluorophore, while more abundant markers work with dimmer fluorophores. We paired three membrane markers, CD4, PD-1, and ICOS, with high and medium fluorophores without spectral bleeding in the panel. This design allows us to minimize the quantity of Opal deposition while maintaining balanced signals and thereby maximally reducing tyrosine depletion or a potential umbrella effect. Misinterpretation due to spectral bleeding between Opal 540/570 and Opal 650/690, which are frequently observed with the Vectra Polaris scanner, can be avoided by selecting markers that are not expressed in the same cellular compartment such as CD20-Opal 540 and Ki67-Opal 570 or FOXP3-Opal 570. To fix the Opal pairing, we determined the Opal intensity count (OIC) for each combination using the InForm software count tool and plotting the autoexposure time and signal-to-noise ratio (Figure 3A). The lowest autoexposure time and highest signal-to-noise ratio are highly recommended, which is why we decided to pair ICOS with Opal 520 and PD-1 with Opal 650. Alternatively, the pairing of CD4 with Opal 620 was not optimal at a low OIC, high autoexposure time and low signal-to-noise ratio; therefore, we used a secondary HRP antibody from Dako (EnVision^+^ System-HRP Labelled Polymer Anti-Rabbit) to address this issue. Finally, to adjust Opal intensity levels, Akoya recommends a signal-to-noise ratio >10 with an OIC between 5 and 20 and an autoexposure time <150 m s. Based on Akoya’s recommendations, a first monoplex adjustment was performed by testing dilutions of Opal 520 and 650. Optimized monoplex stainings visualized as simulated DAB IHC images within the same InForm project detected no spectral bleeding (no false staining) in the other Opal channels for all markers tested (Figure 3B).

**TABLE 1:** Antibody-Opal pairing strategy.

Opal	Opal brightness rankings	Spectral bleed	Initial pairing
Opal 520	Highest		PD1/CD4/ICOS
Opal 540	Medium		CD20
Opal 570	Medium		Ki67/FOXP3
Opal 620	Medium		PD1/CD4/ICOS
Opal 650	Highest		PD1/CD4/ICOS
Opal 690	Lowest		Ki67/FOXP3

The table shows the strategy used for antibody-Opal pairing. The co-localized surface markers (PD-1, CD4 and ICOS) were associated with the brightest Opals using the rankings on the Vectra Polaris scanner. The markers not expressed in the same cellular compartment were associated with Opals that are more subject to spectral bleed with the Vectra Polaris scanner (black boxes).

#### Staining Order

**FIGURE 4:**
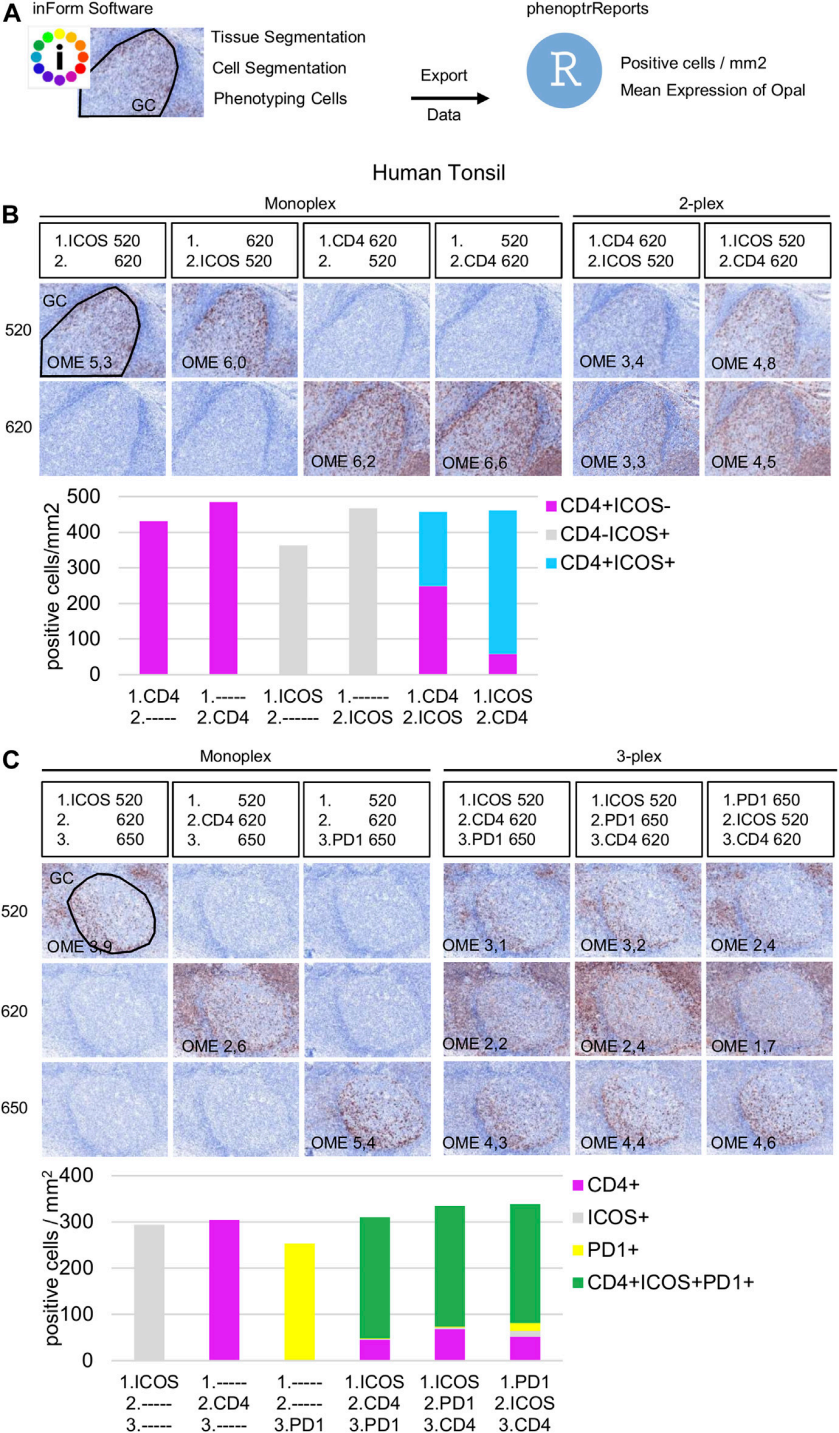
Staining order optimization of three markers co-localized on the cell surface. **(A)** Workflow used to segment tissue regions, segment cells, and phenotype cells with the InForm software before quantifying cell density (positive cells/mm2) and Opal mean expression (OME) using PhenoptrReports on a germinal center (black line). **(B)** Simulated DAB IHC images of monoplex and 2-plex slides staining of consecutive FFPE tonsil sections alternating the position of CD4 and ICOS. The cell densities of CD4^+^ICOS^−^ (magenta), CD4^−^ICOS^+^ (grey), and CD4^+^ICOS^+^ (cyan) were quantified in the monoplex and 2-plex slides. **(C)** Simulated DAB IHC images of monoplex and 3-plex slides stained on consecutive FFPE tonsil sections, including PD-1 in first, second, or third position in the ICOS > CD4 staining order. The cell densities of total CD4^+^ (magenta), total ICOS^+^ (grey), total PD1^+^ (yellow), and CD4^+^ICOS^+^PD-1^+^ (green) were quantified in the monoplex and 3-plex slides. The slides were scanned at ×20 magnification with the Vectra Polaris and the composite images were analyzed with InForm software (v.2.4.8) and PhenoptrReports (Kent S Johnson (2020). phenoptr: inForm Helper Functions. R package v.0.2.6. https://akoyabio.github.io/phenoptr/).

Once the Opal pairing and preliminary signal balancing are completed, a staining sequence based on the epitope sensitivity and stripping efficiency of each primary antibody must be defined. When a given mIHC panel targets co-localized markers (i.e., in the same cellular compartment), the order of staining also must be defined by evaluating TSA interference. The optimization for three markers that can be co-expressed on the cell membrane, ICOS, PD-1, and CD4, is used here as an example. The impact of TSA interference (umbrella effect and/or tyrosine depletion) on multiplex staining was evaluated for monoplex and multiplex slides using sequential tonsil sections stained in different orders, visualized, and then analyzed using InForm and phenoptrReports. The number of detectable cells was determined for the same germinal center (GC) on both monoplex and multiplex slides. For each multispectral image, tissue segmentation, cell segmentation, and cell phenotyping were used to identify and quantify the cell density (positive cells/mm^2^) and the Opal mean expression (OME) for each GC marker (Figure 4A). GCs, transient structures that form in secondary and tertiary lymphoid structures (tonsils and BC, respectively), were selected for quantification because T_FH_ cells (PD-1^+^ICOS^+^CD4^+^) principally reside there and variation in cell density or OME from TSA interference was more easily detected at this site.

Potential interference between co-localized CD4 and ICOS was first analyzed in consecutive FFPE tonsil sections (Figure 4B). Cell density quantification on the 2-plex slides revealed a reduction of ICOS^+^CD4^+^ cell densities associated with increased CD4^+^ cell density when CD4 was stained before ICOS (CD4>ICOS) compared with the inverse (ICOS > CD4). The density of total ICOS^+^ cells in the 2-plex ICOS > CD4 slide was similar to the monoplex slide, but this was not true for the 2-plex CD4>ICOS slide, while the densities of total CD4^+^ cells in both 2-plex slides were similar to the monoplex slides. Even if ICOS^+^CD4^+^ cells are detectable on the 2-plex slides independent of the staining order, this result suggests that Opal 620 deposition on the CD4 epitope partially masks the recognition site of the ICOS epitope when CD4 and ICOS are on the membrane of the same cell. Moreover, in the 2-plex slides, there is a greater reduction in OME 520 when CD4 is stained first, confirming that TSA interference occurs due to CD4-Opal 620 (Figure 4B). Reduction of OME 520 can be fixed by the increasing Opal 520 concentration but the ICOS^+^ CD4^+^ cell density reduction can be only resolved by staining ICOS before CD4 in the multiplex sequence.

Slides were next stained in 3-plex to optimize the staining order for PD-1 in the ICOS > CD4 sequence (Figure 4C). Multiplexed slides reveal that staining PD-1 in the first position increases PD-1^+^ cell density and decreases OME 520 and 620 (ICOS and CD4, respectively) compared to other staining orders. Based on these data we chose ICOS > CD4 > PD-1 for the staining order.

#### Signal Balance Assessment and Crosstalk Interference

**FIGURE 5:**
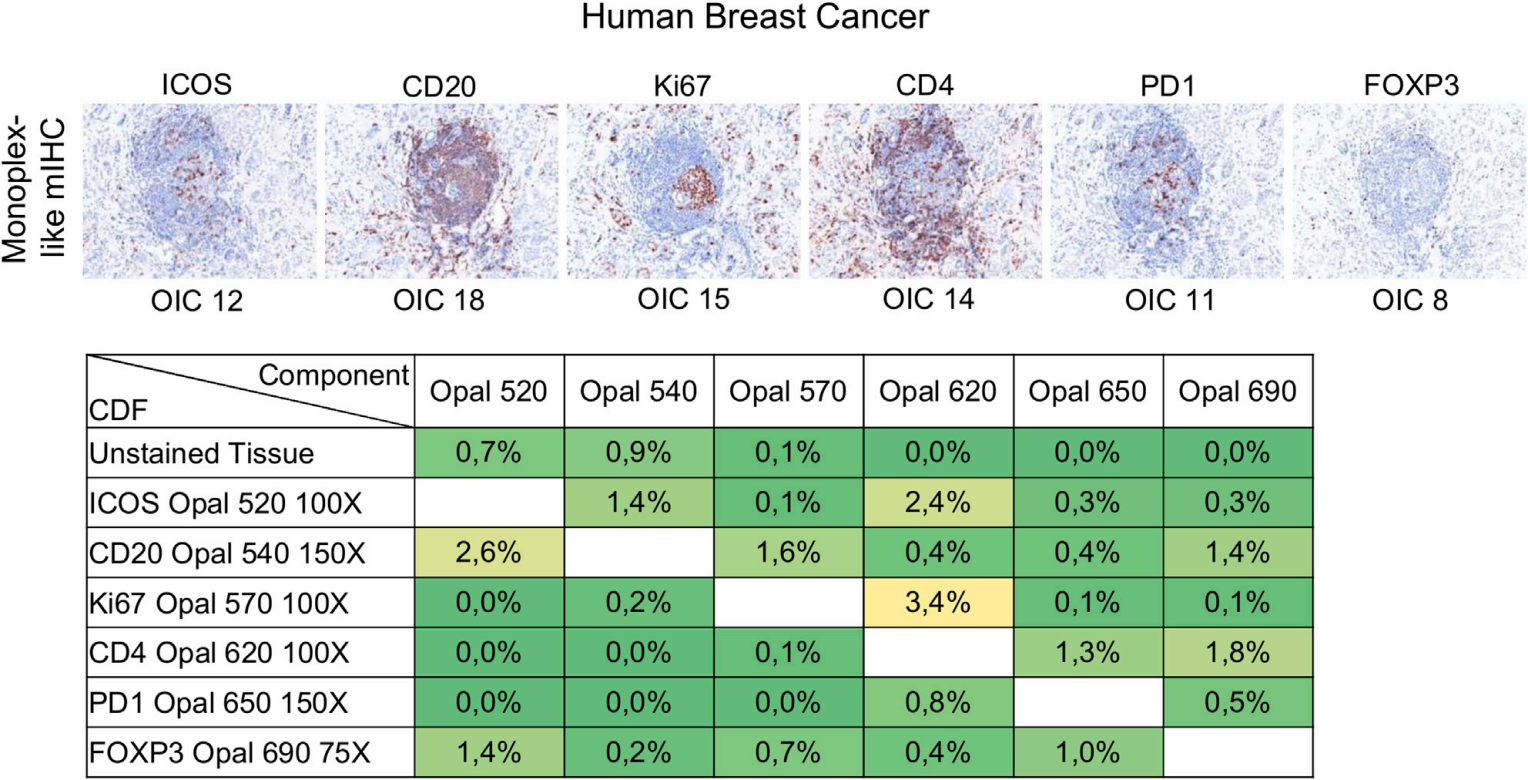
Evaluation of signal balance and crosstalk interference. Simulated DAB IHC images of monoplex stained slides of FFPE breast cancer sections to look like an mIHC slide by replacing the other primary antibodies with diluent. The unmixing quality report table shows the crosstalk from non-signal Opals for each component. The crosstalk values for a component are acceptable for phenotype classification and expression level assessment if all percentages in the component’s column are <5%. The slides were scanned at ×20 magnification with the Vectra Polaris and the composite images were analyzed with InForm software (v.2.4.8) and PhenoptrReports (Kent S Johnson (2020). phenoptr: inForm Helper Functions. R package v.0.2.6. https://akoyabio.github.io/phenoptr/).

Signal levels should be within a factor of three between one another to minimize interference, particularly for spectrally adjacent fluorophores. Evaluation of signal balance is achieved by starting with tonsil monoplex slides, the OIC tool in InForm, and the unmixing quality report in phenoptrReports. Akoya recommends starting the optimization with an Opal concentration of 100X and then adjusting fluorescent intensity signals by increasing or decreasing Opal concentrations. If signals are still too weak, different primary or secondary antibodies can be used or the Opal pairing, additional or more aggressive antigen retrieval methods, and longer primary or secondary antibody incubation times can also be tested. Tumor tissues show variable and heterogeneous target expression compared with tonsils (or other secondary lymphoid tissues) so it is important to evaluate the staining procedure in the target tissue. Monoplex BC slides were subjected to cycles of microwave treatment to simulate multiplex staining and then analyzed by InForm to determine the OIC for each Opal and phenoptrReports to determine crosstalk using the unmixing quality report tool (Figure 5). In contrast to tonsil tissue, ICOS and PD-1 are expressed at lower levels in tumor tissues. Opal 520 and 650 therefore needed to be increased to 100X and 150X, respectively, to restore signal balancing between all of the Opals. Increasing the Opal concentrations for co-localized markers can produce new TSA interference; therefore, it is better to optimize directly using the tissue of interest unless there are limited amounts of these tissues available. The development and validation of an mIHC panel with six markers plus DAPI require a minimum of 70 (4 µm) tissue sections so it is best to use a surrogate tissue if possible when the target tissue is a valuable resource. The Opal dilutions were also adjusted for Opal 540 and Opal 690 so that they were within the target brightness range required. The table of crosstalk by component revealed 2.6% crosstalk for CD20-Opal 540 in ICOS-Opal 520 and 3.4% crosstalk for Ki67-Opal 570 in CD4-Opal 620, both due to spectral bleeding or an unmixing error. 2.4% crosstalk for ICOS-Opal 520 in CD4-Opal 620 was also detected, which was not due to spectral bleeding because Opal 520 and 620 are spectrally distinct. CD4-Opal 620 follows ICOS-Opal 520 staining in the multiplex sequence suggesting that this crosstalk results from inadequate stripping of the ICOS antibody. However, crosstalk values below 5% are acceptable for phenotypic classification and expression level assessment.

#### Multiplex Assay Development

**FIGURE 6:**
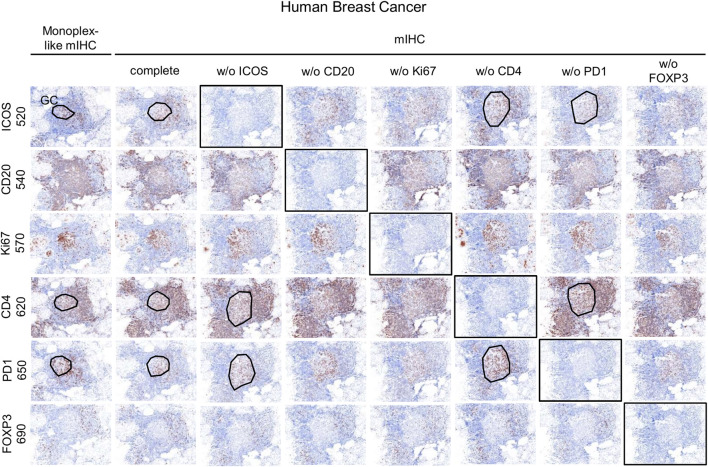
Evaluation of the mIHC using drop controls. Simulated DAB IHC images of monoplex, mIHC, and drop control slides stained on consecutive FFPE breast tumor sections.

**FIGURE 7:**
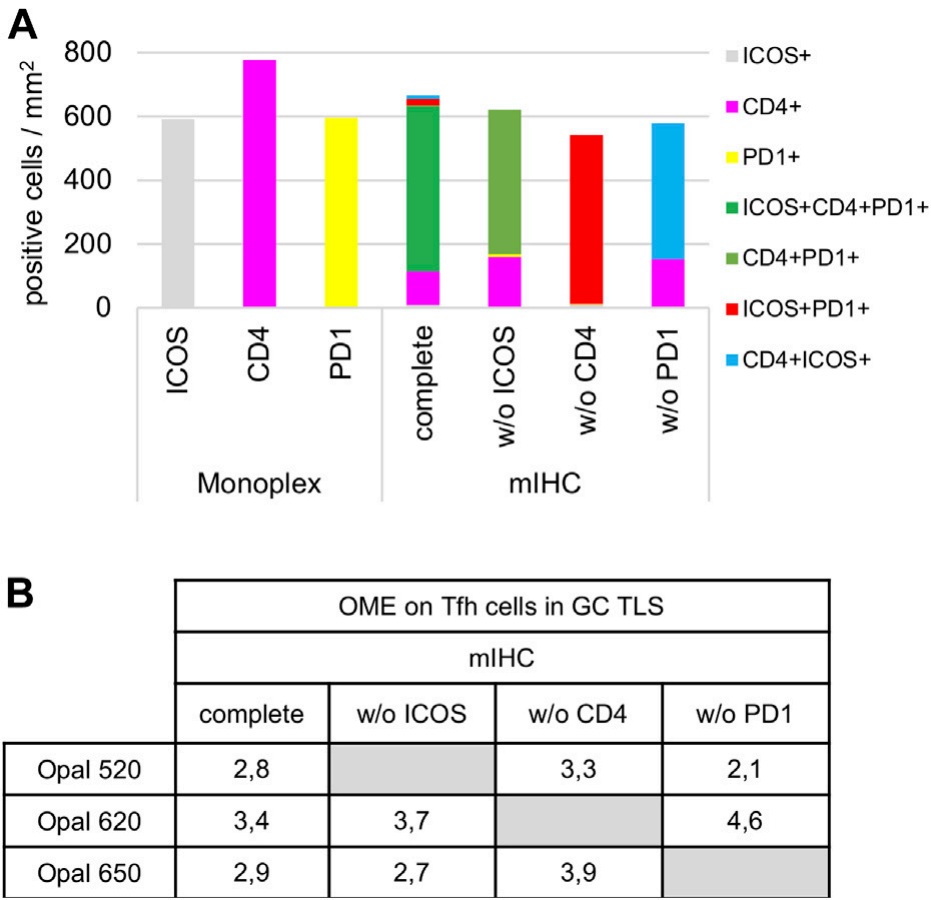
Cell density and Opal expression quantification of drop control slides. **(A)** Cell density quantification of CD4^+^, ICOS^+^, PD-1^+^, and T_FH_ subpopulations (ICOS^+^CD4^+^PD-1^+^, CD4^+^PD-1^+^, ICOS^+^PD-1^+^, CD4^+^ICOS^+^) on monoplex slides, the mIHC slide and drop control slides without (w/o) ICOS, CD4, or PD-1. Each condition was quantified following the workflow shown in Figure 4A in a TLS GC (black dotted line in Figure 6). **(B)** Quantification of 520, 620, and 650 Opal mean expression (OME) on T_FH_ cells from an mIHC slide and drop control slides without (w/o) ICOS, CD4, PD-1 staining. Each condition was quantified followed the workflow as described above. The slides were scanned at ×20 magnification with the Vectra Polaris and the composite images were analyzed with InForm software (v.2.4.8).

Once the monoplex slides were optimized, we looked for potential artifacts in the multiplex staining, such as spectral bleeding and TSA interference. The monoplex and drop controls were compared with the full multiplex panel (Figures 6, 7). The drop controls were identical to the full multiplex except for the absence of one primary antibody in each control slide (Surace et al., 2019). Ideally, each drop control should generate no signal in the dropped channel and no changes in intensity and cell densities. We validated the absence of a signal in all dropped channels (Figure 6). To validate the staining on T_FH_ cells co-expressing CD4, PD-1, and ICOS, cell density and OME were determined within the TLS GC (black line) using the InForm and phenoptrReports software (Figures 7A,B). We observed no significant changes in cell density in the drop controls compared to the full multiplex panel. Alternatively, for T_FH_ cells in the GC of a TLS, the absence of CD4 increased the OME 520 and 650 (ICOS and PD-1, respectively) while the absence of PD-1 increased the OME 620 (CD4) and consequently decreased the OME 520 (ICOS). These data show an absence of spectral bleed in all Opal channels and weak TSA interference on T_FH_ cells due to Opal 620 and Opal 650 deposition, which impacts the Opal intensities without affecting T_FH_ cell density quantification. As an example, Opal 620 intensity levels on CD4^+^ cells and PD-1^+^CD4^+^ cells should not be compared because PD-1-Opal 650 staining leads to reduced CD4-Opal 620 intensities from TSA interference, even if CD4 was stained prior to PD-1 in the mIHC panel sequence. As illustrated in Figure 2D, CD4 intensity is higher at the TLS border than in the GC due to the absence of PD-1 co-expression on these CD4^+^ T cells. This OME variation is due to TSA interference and highlights the fact that marker intensity level quantification on different cell phenotypes requires robust validation.

### TIME Evaluation From Frozen Specimens

While FFPE blocks remain the most readily available source for investigating the TIME, frozen specimens are increasingly being archived to use for staining, spatial transcriptomics (ST), or other approaches where the fixation process destroys or masks some epitopes. Frozen tissues can therefore be used to examine more labile factors and their spatial relationships within the TIME as well as identifying new biomarkers. The main advantages of frozen tissue sections include the preservation of proteins in their native state, which permits a faster staining protocol without the retrieval step and their reliability for molecular analyses such as DNA and RNA sequencing.

#### Immunofluorescence/Immunohistochemistry

Using frozen tissue sections does not require any pre-treatment including deparaffinization, hydration, and antigen retrieval; however, an additional step of fixation should be added before staining. The optimal fixative for the selected target tissue should be determined from a group that includes cold acetone, methanol, or 4% formaldehyde. mIHC using TSA amplification is not recommended for frozen tissue sections because microwave treatments will destroy tissue morphology. Using HRP blocking reagents to quash peroxidase can be an alternative to microwave treatment.

#### Spatial Transcriptomics

Despite recent technological advances in mIHC/IF, the number of markers that can be simultaneously detected is limited, particularly when compared with genomic techniques. Next-generation sequencing enables high throughput whole-genome or whole-transcriptome sequencing; however, these approaches do not provide spatial information. These limitations are being circumvented by ST, an emerging approach designed to transcriptionally profile spatial relationships in gene expression patterns using cancer tissues, with one example being the recent Visium platform introduced by 10X Genomics. This technique provides quantitative visualization and transcriptome analysis using intact fresh-frozen tissues sections and spatially barcoded oligo-deoxythymidine microarrays (Stahl et al., 2016; Vickovic et al., 2019). Following cDNA synthesis, the resulting barcoded cDNA libraries are sequenced using standard RNA-seq technologies. Unique barcodes (UMIs) are used to assign expression data to specific positions on the slide.

**FIGURE 8:**
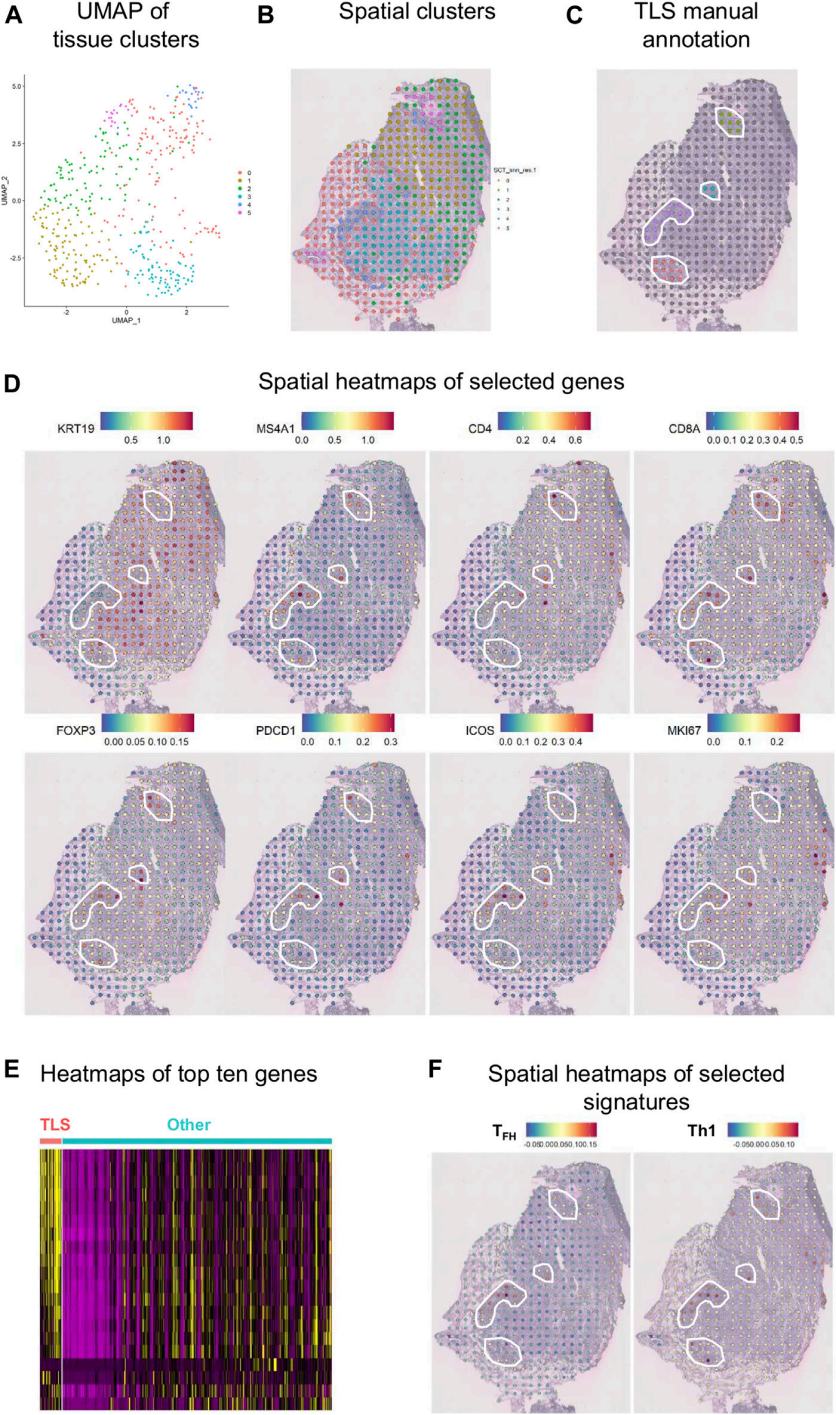
Spatial gene expression in breast cancer. **(A)** UMAP representation of global gene expression in individual spots from a fresh-frozen section of an invasive lobular carcinoma created five distinct clusters with unbiased Seurat clustering. **(B)** The features were placed back onto the H&E staining. **(C)** Four TLS were histologically annotated on brightfield images of H&E-stained tissue sections (white line). **(D)** Visualization of eight selected genes as spatial heatmaps. **(E)** A heatmap of the top ten differentially expressed genes in the annotated TLS compared with the remaining tumor tissue. **(F)** Spatial heatmaps of T_FH_ and Th1 signatures.

An initial examination of the spatial relationship for immune genes in human BC was achieved using the ST technology on a fresh-frozen section from an invasive lobular carcinoma (Figure 8). Using the Seurat algorithm, an open-source R toolkit for single-cell genomics, five clusters were drawn using non-linear dimension reduction (UMAP) in BC (Figures 8A,B) (Butler et al., 2018; Stuart et al., 2019). Interestingly, by superimposing the clusters onto the histological tissue image, an overlap between cluster four and an annotated TLS (based on H&E staining) was observed (Figure 8C). Spatial heatmaps confirmed the expression of immune markers previously identified by flow cytometry and mIHC within TLS such as *MS4A1* encoding CD20, *CD4*, and *CD8A*, as well as their immune activity with the expression of *FOXP3*, *PDCD1* encoding PD-1, and *ICOS* (Figure 8D). Next, we performed a heatmap of the top ten differentially expressed genes in TLS, defined by manual annotation, versus the remaining tumor tissue (Figure 8E). These data identify common gene expression profiles between TLS that differ from the expression profiles of the remaining tumor tissue. Finally, we selected two different immune cell signatures; the T_FH_ and Th1 signatures previously described by our group (Gu-Trantien et al., 2013), to portray the relative enrichment of T_FH_ and Th1 cells within the tumor microenvironment (Figure 8F). The T_FH_ signature was found to be intermediately expressed in two of the four annotated TLS and highly expressed in one, while the Th1 signature was expressed highly in all TLS. These results corroborate previous data from flow cytometry and mIHC, while giving new insights on the spatial distribution.

#### Challenges

Some of the disadvantages of frozen tissue samples are the pre-analytical variables such as time of collection, preservation, and storage in the −80 °C freezer. Tissues need to be frozen as fast as possible once the sample is collected. Moreover, frozen tissue samples rapidly deteriorate at room temperature. The tissue histological quality is lower compared with FFPE samples, and tissues that are frozen incorrectly can form vacuoles. For these reasons, frozen tissue collections remain a smaller component of tumor banks compared to FFPE samples. Coupled with sampling restrictions, one of the major limitations of ST technology is its 50–100 µm resolution, even though the Visium platform expands the spatial resolution 5-fold beyond the first-generation of ST. The recent development of Slide-seq, a method for transferring RNA from tissue sections onto a surface covered in 10 µm DNA-barcoded beads with known positions, overcomes this limitation with a single-cell resolution and holds great promise for the future (Rodriques et al., 2019).

## Discussion

Overall, there are a variety of methodologies that can be used to explore the TIME for discovering the important cellular relationships or identifying relevant biomarkers, but each has distinct advantages and disadvantages. Many of these approaches are considered complementary to one another. In this manuscript, we detailed a number of experimental approaches that are particularly apt for investigating tumor tissues, focusing on TILs and TLS including T_FH_ and T_FR_ cells resident in BC-associated TLS. We further demonstrated it is possible to validate an mIHC panel that includes three co-localized surface markers. Using our rapid and simple non-enzymatic tissue dissociation protocol (Garaud et al., 2014), fresh tissue specimens can be used for TIL phenotyping, analysis of immune soluble mediators, and other characteristics of the TIME (Table 2). Flow cytometry, designed for the analysis of co-expressed markers on single cells, can be standardized for routine analysis and is relatively inexpensive. Because flow cytometric analyses do not provide spatial data, complementary approaches such as IHC/IF must be performed in parallel to obtain this information. cIHC is a useful tool, well established in routine clinical practice, and useful for characterizing individual markers and gaining spatial information on their location in the TIME. The main advantage of cIHC is its use to stain sections from FFPE blocks and their ready visualization with a brightfield microscope, something that is practicable in most pathology labs. In contrast to flow cytometry, cIHC is not capable of staining multiple markers to examine their co-localization. Two markers, usually on distinct cellular subpopulations or different subcellular locations, can be labeled on the same section in experienced labs while three markers are quite rare, which is why cIHC is not recommended when tissue is limited. The development of mIHC, using the TSA technology, has been driven by the need to circumvent sample limitation and the demand for spatial relationship information in a single tissue section. mIHC can presently detect up to eight markers on the same FFPE section. Image analysis software, designed to analyze these fluorescently labeled tissues, is an excellent tool to help scientists and pathologists examine complex cellular phenotypes in a spatial context. The downside of multiplex panel development is that it is time-consuming and requires a dedicated scientist to oversee the efforts and the detection of marker co-expression can be challenging. Despite that caveat, mIHC has emerged as a powerful tool for biomarker discovery and its continued evolution is likely to take it into routine clinical practice in the not-so-distant future. This will require careful assay optimization and validation to ensure robust and reproducible data across laboratories. Moreover, the specificity and sensitivity of mIHC still need to be validated along with consistency between those analyzing the images and need to have experienced pathologists, immunologists, and image analysis experts working together. Finally, while ST technology is a look into a future with full transcriptome analysis in whole tissue sections, major technical limitations do not accommodate its current use in clinical practice. These include the need for fresh-frozen tissues, single-cell spatial resolution that is not yet achieved, lower sensitivity compared with classical *in situ* hybridization analysis, high costs, and the required bio-informatics expertise.

**TABLE 2:** Summary of technologies to investigate the TIME.

Method	Flow cytometry	Chromogenic IHC	Multiplex IHC	Spatial transcriptomics
Sample type	Fresh/frozen cells	FFPE/frozen tissue	FFPE tissue	Frozen tissue
Number of markers	18^+^	2^+^	Up to 8	Whole transcriptome
Detection system	Antibody	Enzymatic reaction	Enzymatic reaction	Barcoded primers
Read out	Fluorescent	Chromogenic	Fluorescent	Sequencing
Co-expression	Yes	No	2^+^	Not applicable
Soluble mediators	Yes	No	No	No
Cost	$	$	$$	$$$
Spatial information	No	Yes	Yes	Yes
Observer	Scientist/biologist	Scientist/pathologist	Scientist/pathologist	Bio-informatician
Clinical relevant	No	Yes	Yes	No

## Data Availability

The raw data supporting the conclusions of this article will be made available by the authors, without undue reservation.
